# Microbial protein synthesis, digestible nutrients, and gain weight of Bligon goats receiving total mixed ration based on sorghum silages (*Sorghum bicolor* L. Moench)

**DOI:** 10.5455/javar.2022.i582

**Published:** 2022-05-27

**Authors:** Bambang Suhartanto, Eka Rizky Vury Rahayu, Nafiatul Umami, Dian Astuti

**Affiliations:** 1 Faculty of Animal Science, Universitas Gadjah Mada, Yogyakarta, Indonesia; 2 Agrotecnology Innovation Agriculture Center, Universitas Gadjah Mada, Yogyakarta, Indonesia

**Keywords:** ADWG, microbial protein synthesis, sorghum silage

## Abstract

**Objectives:**

The goal of this research was to figure out the effect of the local sorghum as silage on the performance of Bligon goats. Microbial protein synthesis, digestible nutrients, and average daily weight gain (ADWG) were measured to evaluate the goats’ performance.

**Materials and Methods:**

The study was designed in a completely randomized design with a one-way pattern. Twelve female Bligon goats with 24.33 ± 2.83 kg (mean ± SEM) initial body weight were divided into three groups of total mixed ration (TMR) treatments. Group 1 received fresh Napier grass (FNG) as a control, group 2 received imported sorghum silage (ISS) of brown midrib resistance (BMR), and group 3 received local sorghum silage (LSS) of super-2. Analysis of variance was used to analyze the data on microbial protein synthesis and feed intake during the research. On the contrary, analysis of covariance was used to analyze ADWG with initial weight as a covariate.

**Results:**

Microbial protein synthesis, feed-intake, and ADWG of goats that received TMR based on silage of two varieties of sorghum, namely BMR (ISS) and super-2 (LSS), were lower (*p* < 0.05) than control. However, there was no significant difference between both TMR based on sorghum silages. ISS’s feed conversion was better than LSS (*p* < 0.05), and FNG was the best. Sorghum silage as a basal ration in TMR had lower microbial protein synthesis but higher total digestible nutrient content than fresh forage, such as Napier grass. The sorghum varieties did not affect the microbial protein synthesis, digestible nutrients, and ADWG of Bligon goats. However, ISS treatment had higher feed efficiency than LSS.

**Conclusion:**

The local sorghum (super-2) silage can be used as ruminant feed as well as imported sorghum (BMR) offered as TMR. However, regardless of the cultivar, TMR based on sorghum silage cannot replace TMR based on fresh Napier grass.

## Introduction

Goats are a vital livestock commodity widely developed in Indonesia, either for meat or milk production. One of the most popular breeds is the Bligon for meat production, and this breed originated from Kacang and Etawah crossbred goats. Bligon goats are widely spread in Indonesia, especially in Central Java province [1]. Performance is one of the parameters that must be pursued in raising goats, and it must be supported by providing high-quality feed and being available continuously throughout the year. 

One of the cereal crops usually planted and developed during the dry season for ruminant feed is sorghum (*Sorghum bicolor* L. Moench), especially in marginal land and suboptimal areas in Indonesia. Sorghum is more adaptable to most agro-ecologies, has higher drought tolerance, and produces more biomass than other cereal crops [2,3]. Sorghum can be harvested more than once [4]. Besides playing a role as forage, sorghum generally produces seeds high in protein and starch content. Nevertheless, due to the high lignin content, sorghum has lower digestibility than maize, which restricts its use in ruminant feeding [5,6]. Sorghum forage can be used as roughage in small ruminant feeding at different levels without affecting intake, digestibility, and nitrogen balance [7]. Sorghum forages are usually processed into silage to remove the prussic acid content. During silage making (ensilage), the prussic acid content in sorghum forage can be reduced by more than 70% [8]. A total mixed ration (TMR) is used for feeding cows that combines feeds formulated for a specific nutrient content into a single feed mix. The total mixed ration is prepared to provide sufficient nutrients for beef and breeding cattle, particularly physiological levels that can produce high productivity and give more technically and economically efficient performance [9]. Total mixed rationing ensures an even distribution of daily ratio intake, reducing rumen microbial fluctuations [10]. 

Microbial protein has an important role in ruminant nutrition because it provides 50%–80% of the total absorbed protein. Dietary manipulation to increase microbial protein synthesis is necessary and should be done [11]. Microbial protein synthesis affects feed nutrients in the digestive tract that are expressed by body weight. The amount of microbial protein synthesis in the rumen can be predicted using purine derivative compounds found in urine, namely allantoin, uric acid, xanthine, and hypoxanthine [1]. Body weight gain becomes an important variable in determining the performance of an animal. The quality of feed and its utilization in an animal can be measured by increased body weight. The study about microbial protein synthesis and the average daily gain of Bligon goats with a diet based on silages of sorghum forage in Indonesia is still limited, so it is important to carry out an advanced study about it. The study’s goal was to find out how much microbial protein, digestible nutrients, and weight gain Bligon goats that were fed TMR based on silage of local and imported sorghum gained when they were fed.

## Materials and Methods

The *in vivo* digestibility trial was carried out at the Agro Technology Innovation Center (PIAT) Universitas Gadjah Mada (−7.796^o^ South Latitude, 110.463^o^ East Longitude), Tanjungtirto, Kalitirto, Berbah, Sleman, Yogyakarta, Indonesia. 

### Ethical approval

The ethical committee of Universitas Gadjah Mada approved this study protocol for preclinical trials with reference number 00027/04/LPPT/V/2020.

### Animal

Twelve female Bligon goats, nonpregnant and nonlactating, approximately 12 months old, weighing 24.33 ± 2.83 kg (mean ± SEM) of initial body weight (BW), were randomly assigned to 3 groups consisting of 4 animals in each group and housed in individual pens with dimensions of 70 × 150 cm and had unrestricted access to water (*ad libitum*). Each pen was equipped with a separate fecal and urine collecting bucket.

### Diets

Diets were distributed based on dry matter requirement, about 3% of BW to gain a body weight of 75 gm/day according to the NRC [12], namely CP 83 gm/day and total digestible nutrients (TDN) 0.51 gm/day for the goats’ body weight of 25 kg. Diets were offered twice daily (8 am and 4 pm) in two equal portions. 

### Ensilage

At 60 days, the sorghum forage was collected and chopped into 3–5 cm in length. The withered sorghum forage was added with molasses up to 4% of dry matter and mixed until the material was completely homogenous. The mixture was then fermented in polyvinyl chloride (PVC) drum silos under anaerobic conditions for at least 21 days [13]. The chemical composition of feedstuffs was summarized in Tables 1 and 2 for feed formulation (gm/kg of ration) and chemical composition (gm/kg DM) of TMR sorghum silage in this study.

### In vivo digestibility

The study was carried out for 70 days, consisting of a 28-day adaptation period, a 42-day collection period, including 14 days of feeds, feces, and urine collection, and measuring body weight gain. In vivo digestibility tests were carried out using the total collection method, according to Avornyo et al. [14].

### Adaptation period

Bligon goats were weighed before entering this period to determine their initial body weight, which was then used as a basis for determining feed requirements. The purpose of this period was to get the livestock accustomed to consuming TMR according to the treatment and to eliminate the effects of the last feed. The study applied the deworming agent Leva-200 oral at a dose of 1 cc/20 kg body weight to prevent worm infection during this period.

### Collection period

Goats were fed according to diet treatments, namely, group 1 received fresh Napier grass (FNG) as a control, group 2 received imported sorghum silage (ISS) of brown midrib resistance (BMR), and group 3 received local sorghum silage (LSS) of super-2. Feed was offered twice daily at 8 am and 4 pm, and the residual feed was collected at 7 am the next day. The feed sample was taken daily until the end of the collection period. The feed sample was then composited and ready to be analyzed. Feces were separated from urine and collected daily. Feces were collected, weighed, mixed, and then the fecal sample was taken proportionally as much as 10% of the daily fecal production. The sample was drained, ground, and composted for further analysis. The urine excreted for 24 h by each animal was collected using a plastic bucket filtered to separate contaminants from the urine. The urine samples were taken as of 10% of the total urine excreted and 10% sulfuric acid was added until the urine pH fell below 3. 

**Table 1.** Chemical composition of feedstuff.

Feedstuff (%)	DM	OM	CP	EE	CF
Napier grass in fresh	10.73	81.36	9.33	1.90	30.48
Imported sorghum silage (BMR)	22.94	89.67	7.12	2.90	25.91
Local sorghum silage (super-2)	19.07	87.72	4.92	2.51	30.95
Imported sorghum (BMR) in fresh	21.86	89.25	7.77	2.54	27.17
Local sorghum (super-2) in fresh	23.62	89.44	5.00	2.48	33.68
Husk of soybean	89.83	94.29	9.53	1.74	37.70
Palm kernel meal	91.48	92.74	18.63	8.24	10.66
Wheat pollard	90.50	94.79	13.27	2.16	12.90
Rice bran	92.79	81.21	7.34	0.88	43.84

DM = dry matter, OM = organic matter, CP = crude protein, EE = extract ether, and CF = crude fiber.

**Table 2.** Feed formulation (gm/kg of ration) and chemical composition (gm/kg DM) of TMR based on sorghum silage.

Feedstuff (gm/kg of ration)	Based on TMR sorghum silage		
	FNG	ISS	LSS
Napier grass in fresh	600	-	-
Imported sorghum silage (BMR)	-	600	-
Local sorghum silage (super-2)	-	-	600
Husk of soybean	102	102	102
Palm kernel meal	50	50	50
Wheat pollard	80	80	80
Rice bran	168	168	168
Total	1,000	1,000	1,000
**Nutrient content (gm/kg DM)**			
Dry matter (DM)	430.0	480.0	480.3
Crude protein (CP)	115.4	80.6	87.2
Crude fiber (CF)	347.7	348.6	345.5
Extract ether (EE)	18.0	23.3	29.3
Ash	172.9	121.5	116.6
Nitrogen-free extract (NFE)	346.0	402.0	421.3
Total digestible nutrients (TDN)	536.1	490.1	537.6

FNG = fresh Napier grass, ISS = imported sorghum silage, and LSS = local sorghum silage.

### Weighing the animals

Feed measuring was distributed during the collection. Before being fed, goats were weighed (kilograms) at the beginning of the collection period in the morning. Furthermore, goats’ weight was evaluated weekly to adjust the change in feed offered and to measure daily weight gain and animal growth patterns.

### Analysis laboratory

Each animal’s feed residues and feces samples were dried and crushed with a 0.1 mm screen diameter Wiley mill. The samples were proportionately blended from 14 days of collection sampling (the collection period). Mixed samples were then subjected to proximate analysis according to Association of Official Analytical Chemist’s (AOAC) [15]. The results of this analysis were used to determine the TDN, according to Hartadi et al. [16]. 

### Purine derivatives (PD) in urine used to estimate microbial protein synthesis

Mixed urine samples of each animal were analyzed for PD, including allantoin, uric acid, xanthine, and hypoxanthine. Allantoin levels were assessed according to the procedure proposed by Chen and Gomes [17]. The procedure for determining uric acid levels followed the method recommended by Chen and Gomes [17], which adopted the determination of uric acid levels proposed by Fujihara et al. [18]. The determination of xanthine and hypoxanthine levels used the enzymatic spectrophotometry method recommended by Chen and Gomes [17]. 

### The concentration of purine derivatives

Determination of the PD in Bligon goats was calculated using the following equation [17]:

*Y* = 0.84 *X* + (0.018 *W*^0.75 ^e−^0.25X^)

*Y* = PD concentration (mmol/day)

*X* = microbial purine absorbed

0.84= the recovery of absorbed purine as PD in urine

0.018 = Bligon goats value 

*W*^0.75^= metabolic body weight

e^−0.25^ = endogenous contribution of PD to total excretion

### Intake of digestible organic matter (IDOM) and rumen digestible organic matter (RDOM)

RDOM levels were carried out according to the procedure recommended by Agricultural Research Council (ARC) [19], as follows:

IDOM = organic matter intake – organic matter in fecal

RDOM = IDOM × 0.65

### Supply of microbial protein

Microbial protein supply was obtained from the estimated supply of microbial nitrogen (ESMN). Rumen microbial protein synthesis value was used to measure the efficiency of the supply of microbial protein (ESMP). Determination of the ESMN value was calculated using the following equation [17]: 

ESMN (gm N/kg/day) = 0.727 × Microbial purine absorbed (mmol/day)


$$\mathrm{ESMP}\;(\mathrm{gm}\;\mathrm{NM}/\mathrm{kg}\;\mathrm{DOMR})=\frac{\mathrm{ESMN}\;(\mathrm{gmN}/\;\mathrm{kg}/\;\mathrm{day})}{\mathrm{RDOM}}$$

### Digestible nutrients

Digestible nutrients were calculated based on the difference between the nutrients’ intake and nutrients in the feces. Measurement of digestible nutrients included crude protein, extract ether, crude fiber, and nitrogen-free extract [9], which were used to determine the total digestible nutrients (TDN) values using the following equations proposed by Hartadi et al. [16]:

Digestible nutrients (gm/kg BW^0.75^/day) = Nutrient intake – Nutrient in fecal 

TDN = d-NFE+d-CF+d-CP+2.25(dEE)

Where, d-NFE = Digestible Nutrient Free Extract, d-CF = Digestible Crude Fiber, d-CP = Digestible Crude Protein, dEE = Digestible Extract Ether.

### Average daily weight gain (ADWG)

Average daily weight gain (ADWG) was calculated based on the difference between initial body weight and final body weight divided by the length of maintenance (days). The weighing of Bligon goats was carried out every Sunday in the morning before being fed.


$$\mathrm{ADWG}=\frac{\mathrm{Initial}\;\mathrm{body}\;\mathrm{weight}\;(\mathrm{gm})-\mathrm{Final}\;\mathrm{body}\;\mathrm{weight}\;(\mathrm{gm})}{\mathrm{The}\;\mathrm{length}\;\mathrm{of}\;\mathrm{ma}\mathrm{int}\mathrm{enance}\;(\mathrm{day})}$$

### Feed conversion

Feed conversion was calculated based on nutrient intake and average daily weight gain. 


$$\mathrm{Feed}\;\mathrm{conversion}=\frac{\mathrm{Nutrient}\;\mathrm{int}\mathrm{ake}\;(\mathrm{gm})}{\mathrm{ADWG}\;(\mathrm{gm}/\mathrm{head}/\mathrm{day})}$$

### Statistical analysis 

All results of the data, except ADWG, were subjected to one-way analysis of variance. The data of ADWG was subjected to analysis of covariance with initial body weight as the covariant. The differences between treatment means were subjected to DMRT when *p* < 0.05. SPSS version 23.0 was employed to analyze the data. 

## Results

### Purine derivatives total excretion in the urine

The average urine volume of Bligon goats with the FNG, ISS, and LSS of TMR treatment was 1036.49, 617.47, and 668.98 ml/day, respectively. The ISS and LSS treatments had no significant difference. However, TMR treatment based on sorghum silage decreased urine volume (*p* < 0.05) when compared with FNG.

The average total urine excretion measurement (purine derivatives concentration, feed digestibility, and microbial protein supply) in female Bligon goats was shown in Table 3. The concentration of allantoin after LSS treatment was higher (*p* < 0.05) than ISS but still lower (*p* < 0.05) when compared with FNG as a control. The uric acid content in ISS and LSS treatments had no significant differences (*p* > 0.05), but it was lower than in FNG. Compared to ISS and FNG treatments, the xanthine–hypoxanthine content in the LSS treatment was lower (*p* < 0.05).

The results indicated that there was no statistically significant difference (*p* > 0.05) in the total excretion of purine derivatives and microbial protein supply (EMNS and EMPS) between a comparison of ISS and LSS treatments. Still, it was lower (*p* < 0.05) than the FNG group. The treatment of ISS and LSS on the digestibility of dry matter and organic matter did not result in a significant difference (*p* > 0.05), but they were higher (*p* < 0.05) than FNG as a control.

### Digestible nutrients

**Figure 1.**
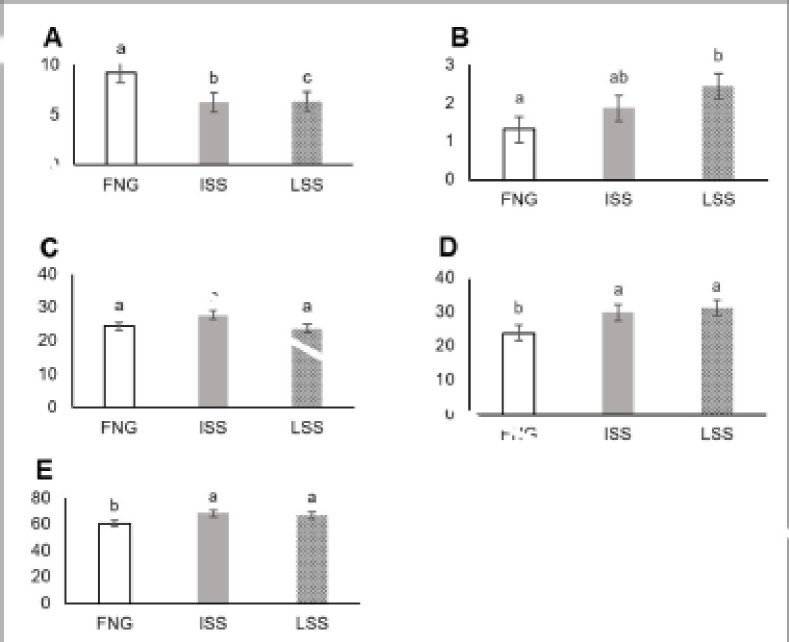
Digestible nutrient of Bligon goats with diet based on sorghum TMR (gm/kg BW^0.75^/day). (A) digestible crude protein, (B) digestible extract ether, (C) digestible crude fiber, (D) digestible nitrogen-free extract, and (E) total digestible nutrients.

Figure 1 showed the average and total digestible nutrient values obtained in female Bligon goats during the research. The values of digestible protein, digestible extract ether, digestible crude fiber, digestible nitrogen-free extract, and total digestible nutrient value of ISS and LSS treatments did not show a significant difference (*p* > 0.05). The values of digestible nitrogen-free extract and total digestible nutrients in ISS and LSS treatments were higher (*p* < 0.05) than those of the FNG group as a control.

### Gain weight and feed conversion

**Figure 2.**
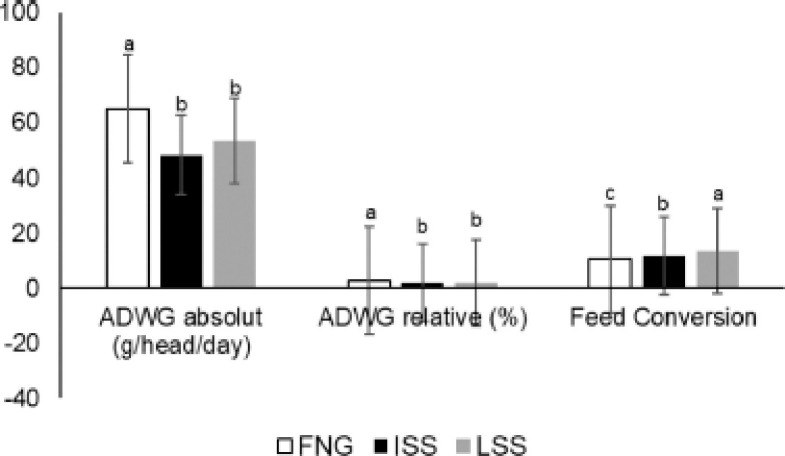
Average daily weight gain (ADWG) absolute, relative, and feed conversion of Bligon goats with diet based on sorghum TMR

Figure 2 showed the average daily weight gain absolute, ADWG relative, and feed conversion of the Bligon goats during the research. The results showed that ISS and LSS treatments resulted in ADWG absolute and ADWG relative, which was not a significant difference (*p* > 0.05). However, it was still lower compared to the FNG group (*p* < 0.05). Compared to ISS and FNG treatments, the feed conversion of the LSS treatment was the highest (*p* < 0.05). 

## Discussion

### PD in the total urine

Allantoin was the largest proportion of total PD. Purwati et al. [20] reported that allantoin excretion in Bligon goats was 54.86–90.46 μmol/BW^0.75^/day. Carro et al. [21] stated that allantoin excretion from purine derivatives in goats is 87.3%–90.8%. The difference in allantoin excretion correlated with energy and protein sources [1]. 

The rumen’s microbial protein production was estimated using the number of PD excreted in the urine. Microbial protein synthesis was essential in ruminants because it provided the host animal with many high-quality protein resources, accounting for 50%–80% of total absorbable protein [22].

The total excretion of PD in TMR based on sorghum silage (ISS and LSS) was low due to the lower feed consumption than FNG. According to Rahayu et al. [13], based on the results of this study, Bligon goats given TMR based on Napier grass fresh forage resulted in a higher nutrient feed intake than BMR or super-2 of sorghum silage. High feed intake positively correlated with increased total excretion of PD and vice versa. Saeed et al. [23] stated that PD excretion was related to dry matter intake [24]. The PD of excretion in the urine is derived from the nucleic acid metabolism of microbes absorbed into the intestines. The metabolic process ran enzymatically, while enzymes were proteins whose synthesis was determined by the DNA of an organic compound that acted as genetic material [20]. Based on the results obtained, the excretion of PD in this study was similar to that reported by Putra et al. [1]. The excretion of PD in Bligon goats fed peanut straw was 114.14 μmol/BW^0.75^/day. In addition, Purwati et al. [20] reported that the excretion of PD in Bligon goats fed *ad libitum* with peanut straw was 72.40 μmol/BW^0.75^/day.

**Table 3.** The concentration of purine derivatives (PD), digestibility, and supply of microbial protein of Bligon goats with diet based on sorghum TMR.

Parameters	TMR based on sorghum silage			SEM	*p*-value
	FNG	ISS	LSS		
**Concentration of purine derivatives (μmol/BW^0,75^/hari)**					
Allantoin	136.90^c^	62.60^a^	72.22^b^	11.55	0.00
Uric acid	34.77^b^	17.27^a^	22.80^a^	3.02	0.02
Xanthine–hypoxanthine	4.65^b^	4.89^b^	3.74^a^	0.21	0.03
Total purine derivatives	176.32^b^	84.78^a^	102.76^a^	14.33	0.00
**Digestibility **(gm/kg BW^0.75^/day)					
IDOM	65.57^a^	72.56^b^	72.46^b^	1.35	0.02
RDOM	42.62^a^	47.17^b^	47.09^b^	0.87	0.02
**Supply of microbial protein**					
ESMN (gm N/kg/day)	1.45^b^	0.68^a^	0.89^a^	0.12	0.00
ESMP(gmNM/kg DOMR)	34.08^b^	14.41^a^	19.01^a^	3.15	0.00

Means with different superscripts in the same row indicate a significant difference (*p* < 0.05). IDOM = Intake of digestible organic matter, RDOM = rumen digestible organic matter, ESMN = estimated supply of microbial nitrogen, and ESMP = efficiency supply of microbial protein.

### IDOM and RDOM

The level of IDOM and RDOM were used to ESMN. Then, the ESMP can be determined. The results of RDOM were obtained from IDOM multiplied by 0.65. IDOM and RDOM digestibility in this study were higher than those reported by Irsyammawati et al. [25]. The proportion of sugarcane silage concentrated in TMR silage 60: 40 showed IDOM 44.58 gm/kg BW^0.75^/day and RDOM 28.98 gm/kg BW^0.75^/day. 

### Microbial protein supply

Based on the concentration of PD, microbial protein supply can be estimated. Microbial protein supply was obtained from the ESMN. Then, rumen microbial protein synthesis data can be used to determine the ESMP by adjusting the estimation results of microbial protein synthesis with RDOM.

Many factors influence microbial protein synthesis and production, including dry matter intake, nitrogen ruminal degradability and carbohydrate sources, rumen dilution rate, mineral content, and other factors [22]. This study’s findings were similar to those reported by Putra et al. [1], who found that the ESMN of a Bligon goat fed peanut straw was 0.825 gm N/day. The ESMP values in this study ranged from 14.41 to 33.08 gm NM/kg RDOM. 

The efficiency of microbial protein synthesis in Bligon goats was 4.61 N/kg RDOM. In addition, rumen microbial protein synthesis was also influenced by nitrogen (N) and energy availability, which can be estimated using the RDOM [25]. The results of this study indicated that the TMR of FNG provided the highest microbial N supply among the other two treatment feeds (ISS and LSS). Fresh forage provides higher nutrients for rumen microbial synthesis than fermented forage (silages). The higher organic matter consumption also influenced the higher microbial protein synthesis. The higher IDOM in TMR based on fresh forage resulted in the fulfillment of microbial nutritional needs for growth and development being met so that microbial protein synthesis could increase [26,27]. According to Gosselink et al. [28], crude protein was an essential nutrient for the production and synthesis of microbial protein, indicated by the availability of nitrogen and adequate sources of energy for rumen microbes, and protein will not be used as an energy source.

### Digestible nutrients

The similar results of ISS and LSS treatments on digestible extract ether, crude fiber, nitrogen-free extract, and total digestible nutrients correlated with the nutrient content of the TMR. The results of this study were as follows: In accordance with Dewi et al.’s [29] study, the dry matter digestibility of ISS was not significant when compared to LSS, at 66.67 and 66.62%, respectively. The treatment of FNG showed higher digestibility of crude protein and extracted ether. It was associated with crude protein and extracted ether from FNG, which was also higher than ISS and LSS. Crude protein digestibility depends on the source and protection of the protein source [30]. The low digestibility of nitrogen-free extract and total digestible nutrient value was related to crude fiber content [31].

### Weight gain

The gain in weight expressed by the ADWG of FNG was higher than other treatments (ISS and LSS). The higher ADWG in FNG was in correlation with feed intake. Rahayu et al. [13] reported that Bligon goats given TMR based on Napier grass fresh forage resulted in a higher nutrient intake than TMR based on BMR or super-2 sorghum silage. This significant variation in BW was affected by the variation in concentrate level and nutrient content in the TMR [7]. Body weight gain was influenced by various factors, such as genetic and environmental factors. Feed quality and quantity were the environmental factors that affected body weight gain because feed nutrient quality and quantity were needed to meet animal requirements. The environmental factors that affected body weight gain were feed quality and quantity because both feed quality and quantity were needed to meet animal needs for growing.

The insignificant difference between ISS and LSS was due to the same nutrient content (Table 2). Another factor that affected ADWG was the utilization of feed nutrients derived from TMR of ISS and LSS. Fermented feed, like silage, will degrade faster in rumen than unfermented feed (FNG/control). It is supported by the results of IDOM and RDOM in Table 3. The faster the rate of feed breakdown in the rumen and the acidic conditions of TMR, the pH (acidity) of the rumen will decrease, which has an impact on the rumen’s microbial population. Rumen microbial protein synthesis will decrease if the synchronization between the N (protein) and carbon (energy) supplies is not balanced. It can be seen from the results of PD in the urine presented in Table 3, which showed that the TMR based on sorghum silage (ISS and LSS) was lower than the control (FNG). Such conditions will affect the utilization of feed nutrients in the body weight expressed through the animals’ body weight gain. According to Santos et al. [32], feed consumption was an important factor affecting performance, with greater consumption leading to more significant body weight gain in goats, averaging 100 gm/day in goats with a body weight of 27.44 kg. It can be affected by how feed is given to animals because it affects how well they can get nutrients from their food, which is why this statement is important.

Setiyawan et al. [30] reported that a female Bligon goats weighing 16 kg, aged 1.5–2 years, showed ADWG of 30.8–61.3 gm/head. Bligon goats fed with complete fermentation feed for 1 week and 2 weeks showed increases in body weight of 3.12 and 3.30 gm/head/day, respectively [33]. Murdjito et al. [34] reported that the ADWG of female Bligon goats was 150 gm/head/day. Suwignyo et al. [35] mentioned that Bligon goats of 12–15 kg body weight given TMR silage showed ADWG of 34.66 gm/head/day. 

### Feed conversion

With regard to treatment based on sorghum silage (ISS and LSS) in feed conversion parameters, FNG showed a significant difference when compared to TMR. It was due to the intake and digestibility of the fermented feed. Rahayu et al. [13] reported in a previous study that a Bligon goat given TMR based on Napier grass forage (Pennisetum purpureum) resulted in higher nutrient feed intake compared to TMR based on BMR or super-2 sorghum silage. Feed conversion showed how much feed was needed to add 1 kg of animal body weight, and a smaller feed conversion value meant that more feed was used efficiently.

Based on the results, the feed conversions from the lowest to the highest were FNG, ISS, and LSS, respectively. Treatments with FNG (fresh forage) exhibited the best feed conversion compared to ISS and LSS. For increasing 1 kg of body weight, the treatment FNG required 10.48 kg of feed, while ISS and LSS required 11.84 and 13.85 kg, respectively. The feed conversion value depends on the quality of the feed distributed. The higher the quality of the nutrients resulted in, the lower the feed conversion. Good-quality feed was needed in a lower quantity than poor-quality feed [36]. The nutrients in the feed will play an essential role in determining the feed conversion value. Increasing body weight requires more building components, namely water, protein, fat, carbohydrates, and minerals. Feed conversion was closely related to production costs [10,37].

The lower feed conversion value of ISS than LSS treatments was lower feed consumption with similar ADWG. Nasiu et al. [38] reported that Bligon goats aged 12–18 months old weighing about 17–20 kg were offered with total mixed rations composed of 65% grass, 20% rice bran, and 15% soybean meal with vitamin E supplementation of 3% CCPO in levels of 0, 200, and 400 mg/kg BW showed feed conversion of 12.00, 9.28, and 8.76, respectively. Low feed conversion indicates the high efficiency of a feed. Two factors determined feed efficiency: feed consumption and body weight gain [39]. 

## Conclusion

It is concluded that the differences in sorghum varieties between ISS and LSS did not affect microbial protein synthesis, digestible nutrients, or the average daily gain of Bligon goats fed with TMR based on sorghum silage. Protein availability from the microbial protein of Bligon goats is better for gaining weight than the energy availability of total digestible nutrients in the feed. A total mixed ration based on LSS (super-2) can be used as ruminant feed as well as ISS (BMR). However, whole-feed-based sorghum silage with any sorghum cultivar cannot replace fresh Napier grass.

## List of abbreviations

TMR, total mixed ration; FNG, fresh Napier grass; ISS, imported sorghum silage; LSS, local sorghum silage; ADWG, average daily weight gain; BMR, brown midrib resistance; cm, centimeter; BW, body weight; PVC, polyvinyl chloride; TDN, total digestible nutrients; N, nitrogen; M, microbial; mmol, millimoles; NFE, nitrogen-free extract; CF, crude fiber; CP, crude protein; EE, extract ether; CCPO, capsulated crude palm oil; IDOM, intake of digestible organic matter; RDOM, rumen digestible organic matter, ESMN, estimated supply of microbial nitrogen; ESMP, efficiency supply of microbial protein; PD, purine derivatives.
